# Temporal Variations of Water Productivity in Irrigated Corn: An Analysis of Factors Influencing Yield and Water Use across Central Nebraska

**DOI:** 10.1371/journal.pone.0161944

**Published:** 2016-08-30

**Authors:** Tony Carr, Haishun Yang, Chittaranjan Ray

**Affiliations:** 1 Nebraska Water Center, Water for Food Global Institute at the University of Nebraska-Lincoln, Lincoln, Nebraska, United States of America; 2 Department of Agronomy and Horticulture, Institute of Agriculture and Natural Resources at the University of Nebraska-Lincoln, Lincoln, Nebraska, United States of America; Montana State University Bozeman, UNITED STATES

## Abstract

Water Productivity (WP) of a crop defines the relationship between the economic or physical yield of the crop and its water use. With this concept it is possible to identify disproportionate water use or water-limited yield gaps and thereby support improvements in agricultural water management. However, too often important qualitative and quantitative environmental factors are not part of a WP analysis and therefore neglect the aspect of maintaining a sustainable agricultural system. In this study, we examine both the physical and economic WP in perspective with temporally changing environmental conditions. The physical WP analysis was performed by comparing simulated maximum attainable corn yields per unit of water using the crop model Hybrid-Maize with observed data from 2005 through 2013 from 108 farm plots in the Central Platte and the Tri Basin Natural Resource Districts of Nebraska. In order to expand the WP analysis on external factors influencing yields, a second model, Maize-N, was used to estimate optimal nitrogen (N)–fertilizer rate for specific fields in the study area. Finally, a vadose zone flow and transport model, HYDRUS-1D for simulating vertical nutrient transport in the soil, was used to estimate locations of nitrogen pulses in the soil profile. The comparison of simulated and observed data revealed that WP was not on an optimal level, mainly due to large amounts of irrigation used in the study area. The further analysis illustrated year-to-year variations of WP during the nine consecutive years, as well as the need to improve fertilizer management to favor WP and environmental quality. In addition, we addressed the negative influence of groundwater depletion on the economic WP through increasing pumping costs. In summary, this study demonstrated that involving temporal variations of WP as well as associated environmental and economic issues can represent a bigger picture of WP that can help to create incentives to sustainably improve agricultural production.

## Introduction

There is much to suggest that pressure on water resources will increase in the future. Forecasts for the year 2050 predict that the world population will increase to approximately 9.15 billion with a simultaneous increase of per capita income to 1.8-fold of the present [1]. This development will result in an increasing demand for food due in part to the higher population forecasts as well as the demand for more affluent diets. To adapt to the changing conditions, grain yields need to be increased further. Although agricultural yields have increased in the recent past, the yearly gains are insufficient to double food production by 2050, which is necessary to meet future demands [2]. Water plays a critical role as an input in agricultural systems, and agricultural usage already places enormous pressure on worldwide water resources. Approximately 70% of world-wide freshwater withdrawals are already used by agriculture. However, due to future development the pressure on water resources will increase by more than 20% over the present level by 2050 [3]. Therefore, it is necessary to combine the intensification of agricultural production with sustainable water management measures to reduce the pressure on water resources.

With the concept of water productivity (WP) it is possible to evaluate the sustainability and efficiency of agricultural water management in terms of “produced yields per unit of water used”, often referred to as “crop per drop”. This measure helps to identify disproportionate water use or water limited yield gaps and thereby support improvements in agricultural water management.

Due to the involvement of a wide range of scientific disciplines in the investigation of the relationship between food production and water consumption, WP can be defined in numerous ways. The most suitable definition of WP for this study is the one of Molden et al. [4], in which WP is defined as the ratio of net benefits from crop, forestry, fishery, livestock, and mixed agricultural systems to the amount of water required to produce those benefits [4]. For physical WP, it is the amount of water used for producing a given quantity of crop. The economic WP is the net monetary value of the crop per unit of water used. The same definition extends to animals, forest products, or fish. Water use is mostly expressed by either evapotranspiration as a measure of water consumption by plants or by the total amount of water applied to the fields, including soil stored water at sowing time, and irrigation and precipitation during the growing season. Conceptually, the former (amount of crop evapotranspiration) may be a better measure of crop water use than the latter as the deep percolation component of water balance is not used for crop growth. However, measuring evapotranspiration or deep percolation requires not only significant investment but also small spatial scales such as plot levels.

Whether the physical amount or the monetary value of yields per unit of water use is being analyzed depends on the purpose of a WP study. In addition, the choice of scale of a WP analysis needs to be considered. This includes plant or plot level, field level or agro-ecological level [5]. Beyond the considerations in respect to the correct definition and scale of a WP analysis, it is also important to consider the variety of factors influencing yields and water use. Often qualitative and quantitative ecological and economic factors are not considered in the equation and therefore restrict the conclusions, which can be drawn from analyzing the productivity of water use.

The different approaches and scales of a WP analysis can lead to different conclusions, but also the many factors influencing yield production and water use can cause varying WP -values over time and space. Several studies have already demonstrated the impact of farmer’s management decisions as well as present soil and spatially different climate conditions on WP [6–10]. The objective of this study is to expand the discussion around factors connected to WP in order to gain a broader picture about their influence on WP. Thus, a more comprehensive picture of WP can be developed for supporting methods to sustainably improve the productivity of water use in agriculture. For this purpose the evaluation of WP in irrigated corn production in central Nebraska, USA will be used as an example. In this respect, the degree of WP will be evaluated at several locations in Nebraska while also considering temporal variations of WP during nine consecutive years in the study area. The temporal dynamics of WP will be analyzed with a focus on the variations of weather conditions during the observation period in the study area. In addition, the impacts of irrigation and fertilizer management on WP in respect to changing weather conditions will be briefly discussed. Furthermore, this study will include environmental quality and economic factors to improve the informative value of the WP analysis. The analysis of WP and its variability in this study is based on the comparison of yield and water use data collected at 108 irrigated farm fields where continuous corn is grown with model simulated WP (estimated as the maximum yield per unit of water), which can be regarded as the optimal WP because it uses the minimum amount of irrigation to create non water stress growth without waste of water.

## Materials and Methods

### Study area

The state of Nebraska is the third largest corn producer in the USA and has the largest area of irrigated agriculture. Irrigation water is mainly pumped from the underlying High Plains Aquifer, which is one of the largest aquifers of the world. In parts of the High Plains Aquifer intensive pumping of irrigation water has already resulted in declining groundwater levels [11]. Thus, it is important for Nebraska to continuously monitor groundwater levels and evaluate the sustainability of agricultural water management.

**Fig 1:**
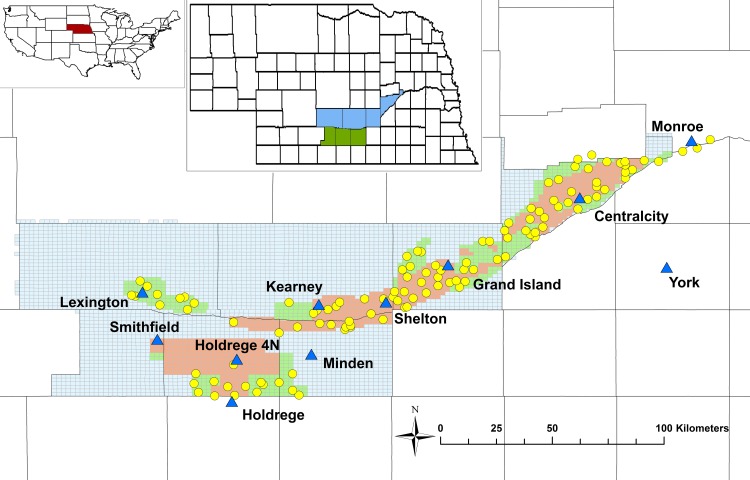
Study area and its location in Nebraska and the USA. Location of Nebraska in the map of the USA (red area in the upper-left map), location of the study area in the Central Platte NRD and the Tri Basin NRD in the map of Nebraska (blue and green areas in upper-middle map), and fields (yellow points) and weather stations (blue triangles) in the study area (main map). The study area in the main map is divided into blue, green and red areas. Those colors represent groundwater quality management areas, which depend on the nitrate concentration in the groundwater. Blue areas in the main map represent phase 1 areas, where nitrate concentrations are acceptable. Green and red areas are phase 2 and phase 3 areas, where nitrate application is regulated by the respective NRD.

Our study was implemented on irrigated corn fields of two Natural Resource Districts (NRD) Central Platte (CP) and Tri Basin (TB) (Fig 1). In summary both NRD’s have an area of 1.3 Mha irrigated crop fields, in which corn is the main crop. The climate in Nebraska varies from a humid continental climate in the east to a semi-arid climate in the west. Annual precipitation in the study area in the years 2005–2013 ranged between 314 mm and 780 mm with an average of 590 mm. The annual maximum temperature ranged between 15.7°C and 19.6°C with an average of 17.1°C and the annual minimum temperature ranged between 2.4°C and 4.1°C with an average of 3.4°C during the same period. The soil in the analyzed locations is predominantly loam.

### Data

The field data for this study were obtained from annual farmers’ reports in the Central Platte and Tri Basin NRDs between 2005 and 2013 submitted to these NRDs as part of the groundwater management program. The annual reports include data of corn yields, irrigation amounts, nitrogen application rates and nitrate concentration in irrigation water on 108 production fields each of which covers 65 ha (160 acres) on average. Irrigation water for each field is mainly delivered from wells pumping the underlying unconfined aquifer, although surface water is also used with the help of a canal supply system. Additional data on water use in the study area was provided by the Central Nebraska Public Power and Irrigation District office in Holdrege, Nebraska. Daily weather data, including maximum and minimum temperature, precipitation, relative humidity, solar radiation and potential evapotranspiration, were retrieved from eleven weather stations in the area for the years 2005 through 2013 [12]. To better represent each field, the weather data from three surrounding stations of each field were interpolated through inverse distance weighting by multiplying each value of one weather station with a weighting factor depending on their distance to the field [13]. The soil properties at each field, such as available water holding capacity, bulk density, soil drainage properties, pH, field slope, soil organic matter, surface and sub-soil texture, were derived with the ArcGIS Soil Data Viewer using the SSURGO 2.2 database [14]. The plant available soil water (PAW) on the planting date at each location and year was calculated by including the plant available soil water holding capacity in the rooting zone, off-season precipitation (October 1–April 30), and residual plant available water of the previous season [13]. Annual crop, fertilizer and energy prices were obtained from the USDA and the Nebraska Energy Office [15, 16]. Data on crop and nutrient management practices, such as the date of planting, hybrid maturity, most commonly used fertilizers, fertilizer application timing and tillage practices were collected in the study of Farmaha et al. [17].

### Modelling software

Three different modelling software packages were used to analyze WP in the study area and chosen drivers influencing yield growth and water use. The software includes a maize growth and yield simulation model, a nitrogen fertilizer rate model for maize, and a water flow and chemical transport model for the unsaturated zone.

The main model used was Hybrid-Maize–available at the University of Nebraska [18–20]. With this crop model the maximum attainable yield was estimated at each location and year (n = 1890) under irrigated and rain-fed conditions. Hybrid-Maize simulates the growth and development of maize under non-limiting or water-limited (irrigated or rain-fed) conditions in relation to weather, soil properties, and management factors. In the simulations of Hybrid-Maize, optimal nutrient supply and no stress due to diseases, insects and pests is assumed. Water is treated as the only limiting factor in Hybrid-Maize.

In order to assess the impact of nitrogen supply on yields and WP in the study area, optimal nitrogen fertilizer rates for the observed yields were simulated, using the crop model Maize-N–also available at the University of Nebraska. Maize-N is built to estimate nitrogen fertilizer requirement for user-specified yield or simulated yield using the built-in Hybrid-Maize routine based on weather, crop management and soil properties. This approach differs from crop models such as DSSAT (Decision Support System for Agrotechnology Transfer) [21] in that it estimates nitrogen fertilizer requirement for a crop yield as determined by factors other than nitrogen, which mimics the decision making process by farmers in the US. The Maize-N simulation results were derived from average data of the years 2005–2013 at each field. Both, Hybrid-Maize and Maize-N, have been validated in irrigated and rain-fed maize systems in Nebraska [22–24].

Solute transport in soils at different locations and years was analyzed with the modelling software HYDRUS-1D in order to demonstrate the impact of leaching processes on nitrate availability as another external factor influencing WP. HYDRUS-1D is a software package for simulating vertical water and solute movement in variably-saturated media [25]. One of the intents of HYDRUS-1D simulations was to determine how weather forcing can impact the movement of nitrogen in the root zone, especially in the early season of plant growth when the root systems are not well developed to use the nitrate in deeper profiles. For clarity, HYDRUS-1D was not coupled to Hybrid-Maize or Maize-N, but it used the same data of weather and soil conditions between 2005 and 2013 that were used for the Hybrid-Maize and Maize-N simulations. Irrigation amounts and timing at the analyzed locations were derived from daily simulations between 2005 and 2013 using Hybrid-Maize. In Hybrid-Maize simulations, irrigation was triggered whenever crop evapotranspiration demand exceeds crop water uptake in the soil, while the irrigation amount is co-determined by user-specified irrigation capacity and root zone soil field capacity. One limitation of using daily time steps for modeling leaching systems was that the rain or irrigation amounts were spread over the entire day (thus reducing their intensity) whereas the actual rain or irrigation may have occurred for a portion of the day. Simulated fertilizer application was set at the beginning of the season on May 1^st^ after a 30-day period of flow-only simulations to neglect the initial soil water regime as an influencing factor on vertical soil transport simulations. One must realize that there are more comprehensive models of nitrogen transport and transformation in agroecological systems (e.g., RZWQM [26]), however, HYDRUS-1D was considered more realistic considering many data for RZWQM will not be available for the simulation sites.

### Evaluation of Water Productivity

In this study, WP was defined by the amount of yield per unit of total water supply including the amount of plant available soil water on the planting date, amount of irrigation and rainfall during the growing season. In a further analysis yield was defined by its net benefit enabling the integration of economic factors in the WP coefficient. This coefficient will be referred to as the economic WP in a brief discussion on the impact of groundwater level changes on energy costs for pumping irrigation water and the net benefit of yield.

WP in the examined locations of the Central Platte and the Tri Basin NRDs was evaluated using the Hybrid-Maize simulation results as a benchmark for an optimal WP in the study area. The results of the Hybrid-Maize simulations were used to compare the observed yields per unit of water supplied to the fields with the simulated maximum attainable yields per unit of water. This comparison was conducted for all 108 locations and for each year between 2005 and 2013 by running Hybrid-Maize with temporal and spatial varying soil and weather conditions. The evaluation of WP in the study area was based on the gap between the observed yields per unit of water and the simulated maximum attainable yields per unit of water at each location and year. As a further analysis of WP, average WP values, expressed through the yield per unit of water supply, as well as the average yields, irrigation water and total water supply of each year was compared between the observed and the simulated data. Thus, year-to-year dynamics of WP were derived for analysis.

### Evaluation of factors influencing Water Productivity

The second step of this study was to examine the factors influencing WP in the study area and evaluate how they contribute to variations of WP values. Yields and water use are influenced by many different factors ranging from farmers’ crop management decisions to weather conditions. The focus of this study was on the latter, since weather has a strong influence on crop growth and water needs and therefore will be used to explain the year-to-year dynamics of WP during the observation period. In this respect, the average annual observed and simulated WP values as well as the average crop yields, amounts of irrigation water and total water supply were compared with annual dynamics of relevant weather variables, such as temperature, precipitation and solar radiation.

In addition to the simulation of the maximum attainable yield per water supply of each season, Hybrid-Maize also generates daily simulation results of crop water consumption and biomass growth. These values were used to analyze the changing seasonal course of crop water stress with respect to the prevailing weather conditions. The simulation results help to determine the impact of weather conditions on crop water needs, which provided the basis on how to adapt irrigation management in order to improve WP.

Fertilizer management as another major factor influencing WP as well as environmental quality was examined with the help of the Maize-N and HYDRUS-1D simulation results. First, the Maize-N simulation results were used to compare the farmer-reported N-fertilizer application rates with the model estimated optimal N-fertilizer application for the observed yields. In this way it was analyzed to what degree a change in fertilizer application rates would impact yield and thus WP of a field. The availability of nutrients in the soil profile delivered through fertilizers further influences yield and WP. Thus, HYDRUS-1D was used to analyze the effect of the degree of precipitation on nitrate leaching on different years in the study area. Because the soil was well drained and warm after fertilizer application, all forms of nitrogen fertilizers are assumed to become nitrate form soon after their applications, while denitrification became negligible [27]. These were considered as reasonable assumptions for our purpose, and the need for running complex geochemical models was eliminated. The results were used to support the discussion about the influence of weather parameters on the variations of WP values.

## Results and Discussion

### Water productivity

**Fig 2:**
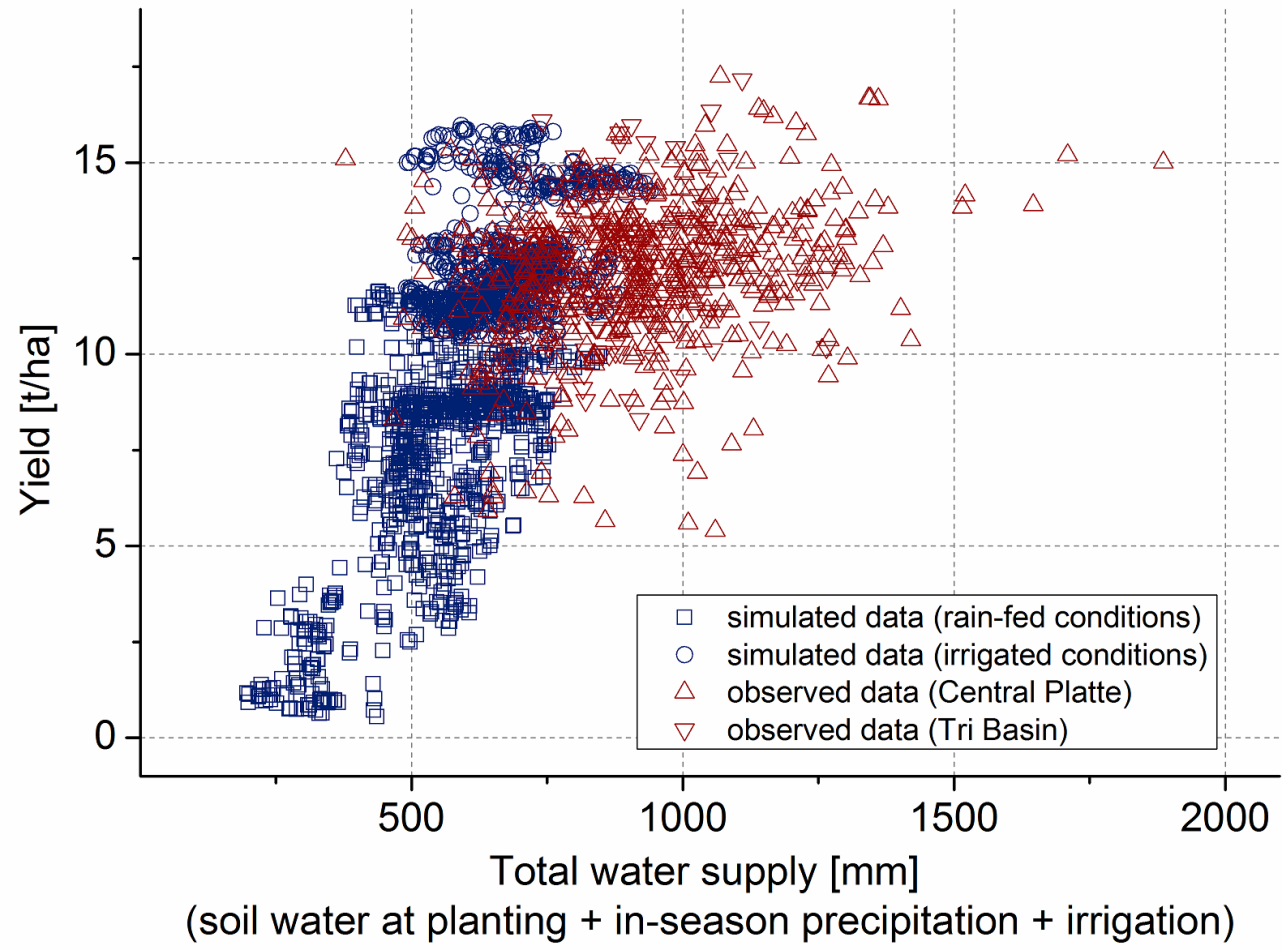
Comparison between observed maize yields and simulated yields for different amounts of water at 108 locations in Nebraska in the years 2005 through 2013. Simulated data are divided between simulation results under rain-fed (n = 945) and irrigated (n = 945) conditions. Observed data are divided between the Central Platte (n = 559) and the Tri Basin (n = 142).

The Hybrid-Maize simulation results of all locations in the Central Platte and in the Tri Basin NRDs under rain-fed and irrigated conditions show a linear correlation between yield and water supply (Fig 2). Minimum, maximum and mean values of the simulated data can be seen in Table 1. Simulation results under irrigated conditions show less variations than simulation results under rain-fed conditions. Additionally, higher yields are achieved under irrigated conditions. The simulated maximum yield is 15.96 t/ha and the maximum amount of total water supply is 940 mm in the Central Platte NRD and 15.59 t/ha and 888 mm in the Tri Basin NRD. The maximum yield values were achieved under irrigated conditions. Simulation results in both NRDs show a similar pattern.

**Table 1:** Simulated minimum, maximum and average maize yields, water supply and WP.

	Central Platte						Tri Basin					
	Rain-fed			Irrigated			Rain-fed			Irrigated		
	Min	Max	Mean	Min	Max	Mean	Min	Max	Mean	Min	Max	Mean
Yield [t/ha]	0.55	11.64	7.17	10.27	15.96	12.46	1.41	11.42	7.52	10.51	15.59	12.57
Total Water Supply [mm]	197	847	526	491	940	664	293	751	574	589	888	715
WP [kg/m³]	0.13	2.85	1.35	1.30	3.05	1.91	0.33	2.06	1.30	1.33	2.51	1.77

The actual data collected from both NRDs are compared with the simulated data in Fig 2. In the collected dataset, 18 outliers outside the range of the triple standard deviation were deleted (S1 Fig). Minimum, maximum, median and mean values of the actual data can be seen in Table 2. A complete overview of the processed data can be seen in S1 Dataset. The maximum reported yield between 2005 and 2013 in the Central Platte NRD and the Tri Basin NRD was 17.25 t/ha and 17.16 t/ha, respectively. Both values are above the maximum simulated yield of 15.96 t/ha. The simulated mean attainable yield under irrigated conditions of 12.46 t/ha in the Central Platte NRD is slightly higher than the actual mean yield of 12.05 t/ha. In the Tri Basin the simulated mean yield of 12.57 t/ha is slightly lower than the actual yield of 12.61 t/ha. Both, simulated and actual values are above the average irrigated corn production rate of 11.77 t/ha in Nebraska during 2005–2013 [15].

**Table 2:** Actual minimum, maximum, median and average data collected in the study area.

	Central Platte				Tri Basin			
	Min	Max	Median	Mean	Min	Max	Median	Mean
Yield [t/ha]	5.41	17.25	12.20	12.05	8.30	17.16	12.73	12.61
Water Supply [mm]	378	1886	911.81	918	640	1266	896.95	905
WP [kg/m³]	0.51	3.99	1.32	1.37	0.81	2.30	1.39	1.42

Unlike the observed and simulated yield, the simulated total crop water and the observed total water supply show large differences. In the Central Platte NRD the maximum amount of total water supply reached a value of 1886 mm in 2012, whereas the maximum simulated amount of total crop water reached only about half as much in the same NRD—940 mm. Also in the Tri Basin NRD maximum total water supply was much higher, 1266 mm in 2007, than the simulated maximum requirement of 888 mm. Average amounts of actual water supply also exceed the average simulated amounts in both NRDs. In the Central Platte the simulated average water requirement of 664 mm is 72% of the observed average water supply of 918 mm. In the Tri Basin the simulated average water requirement of 715 mm is 79% of the observed average water supply of 905 mm.

By dividing the actual yield by the actual amounts of total water supply, the actual WP of each location and year can be calculated. The results vary between 0.51 kg/m³ and 3.99 kg/m³ in the Central Platte and 0.81 kg/m³ and 2.30 kg/m³ in the Tri Basin. The observed mean WP of the Central Platte (1.37 kg/m³) and the Tri Basin (1.42 kg/m³) are below their respective average simulated optima of 1.91 kg/m³ and 1.77 kg/m³, respectively.

The comparison between the observed and simulated data revealed that WP in the study area is not optimal in most cases. The cause for the low WP values in the analyzed locations can be identified after comparing the observed and simulated yields and the water supply amounts. The observed yields are on a similar high level as the simulated yields. In this respect, potential for corn yields in the study area cannot be exploited much further. In contrast to yields, the observed water supply and simulated water requirement show large discrepancy in the majority of cases. The amounts of in-season precipitation and soil water at planting are based on the same calculation and data for both actual and simulated results. Therefore, the large deviation of actual total water supply amounts has to be related to the deviation of actual irrigation amounts from the simulated ones. On average actual irrigation amounts exceed the simulated irrigation requirements with 253 (±189) mm in the Central Platte and 194 (±103) mm in the Tri Basin during the observed nine consecutive years.

### Impact of weather conditions on Water Productivity

**Fig 3:**
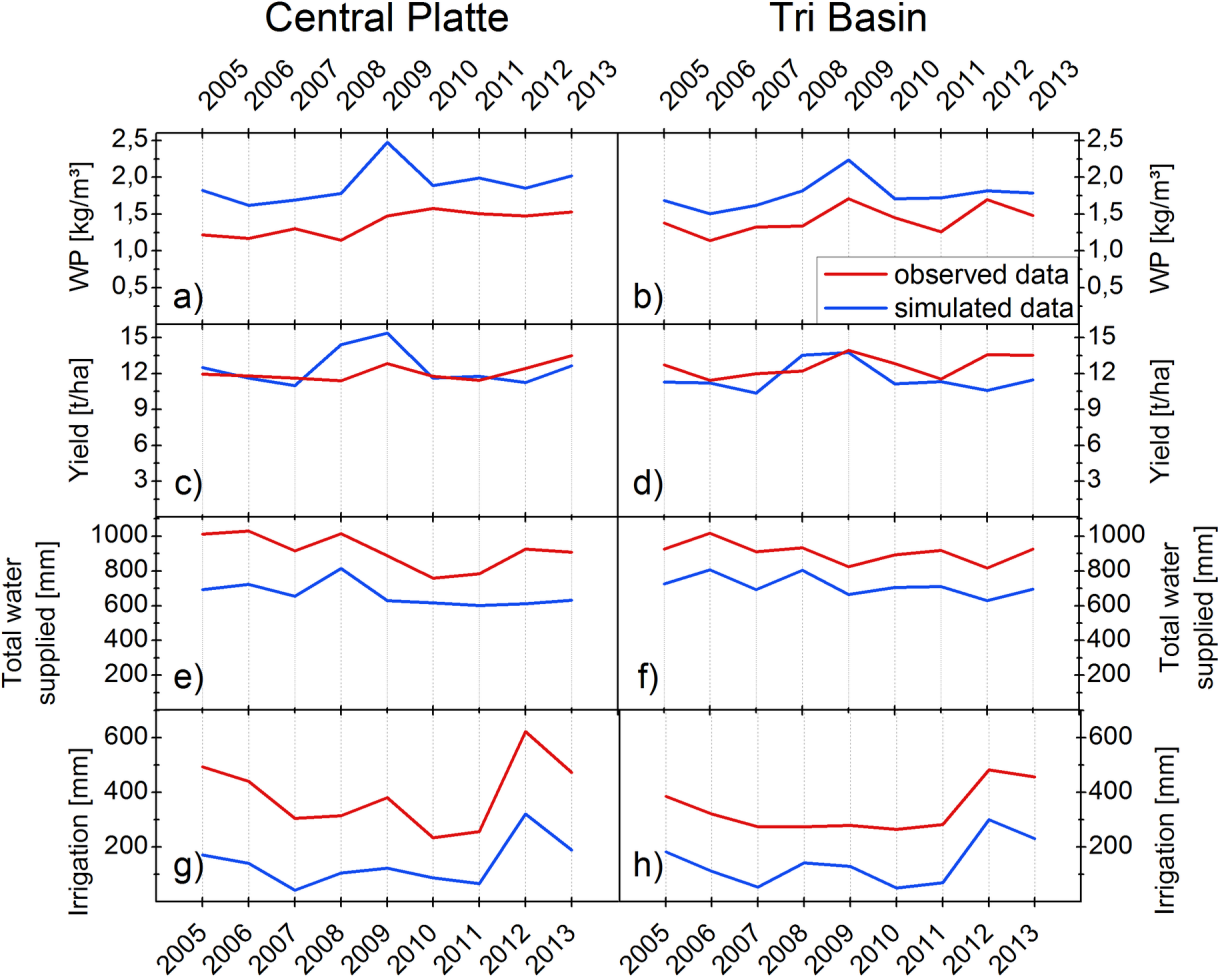
Comparison of annual average simulated and observed data. The graphs illustrate the year-to-year variations in WP (a, b), yield (c, d), amounts of total water supplied (e, f) and irrigation (g, h) as well as the differences between actual and simulated data in the Central Platte and in the Tri Basin between years 2005 and 2013.

The analysis of WP in the study area revealed that in most cases the level of corn yields could be achieved using less irrigation according to the comparison between the simulation results and the observations. Similar conclusions were made in a two-year, large scale study conducted in farmer’s fields across Nebraska by Irmak et al. [28]. In that study, farmers used 32%-34% more irrigation than the necessary amount. The year-to-year average WP, corn yields, water supply and irrigation confirm a potential for improving WP in the study area (Fig 3). Additionally, the annual differences demonstrate the variations of WP. The actual WP from the field data vary from 1.1 kg/m³ to 1.6 kg/m³ in the Central Platte NRD and from 1.1 kg/m³ to 1.7 kg/m³ in the Tri Basin NRD. In the Tri Basin NRD a peak of the WP value can be seen in 2009 and 2012. In the Central Platte NRD it can be observed that for 2008 the average WP is lower compared to all other years.

The variations of WP between 2005 and 2013 can be related to the year-to-year differences of average yield, water supply and irrigation amounts. Actual yield in the Central Platte range from 11.4 t/ha to 13.5 t/ha. The highest yield was achieved in 2013 closely followed by yields in 2009 and 2012. In the Tri Basin NRD actual annual yield rates range from 11.4 t/ha to 13.9 t/ha. The highest yield was achieved in 2009 followed by the yield of 2012 and 2013.

The actual amounts of total water supply in the Central Platte ranged from 758 to 1030 mm. The highest actual amounts of water supply can be observed at the beginning of the observation period. In the Tri Basin NRD actual water supply ranged from 816 to 1017 mm with the highest peaks at the first years of the observation period as well. Irrigation amounts varied more between 2005 and 2013 than did total water supply amounts. In the Central Platte irrigation amounts ranged from 234 mm to 623 mm with the peak in 2012. Also in the Tri Basin the highest irrigation amounts can be observed in 2012 with 483 mm. The minimum irrigation amount in the Tri Basin is 265 mm.

**Fig 4:**
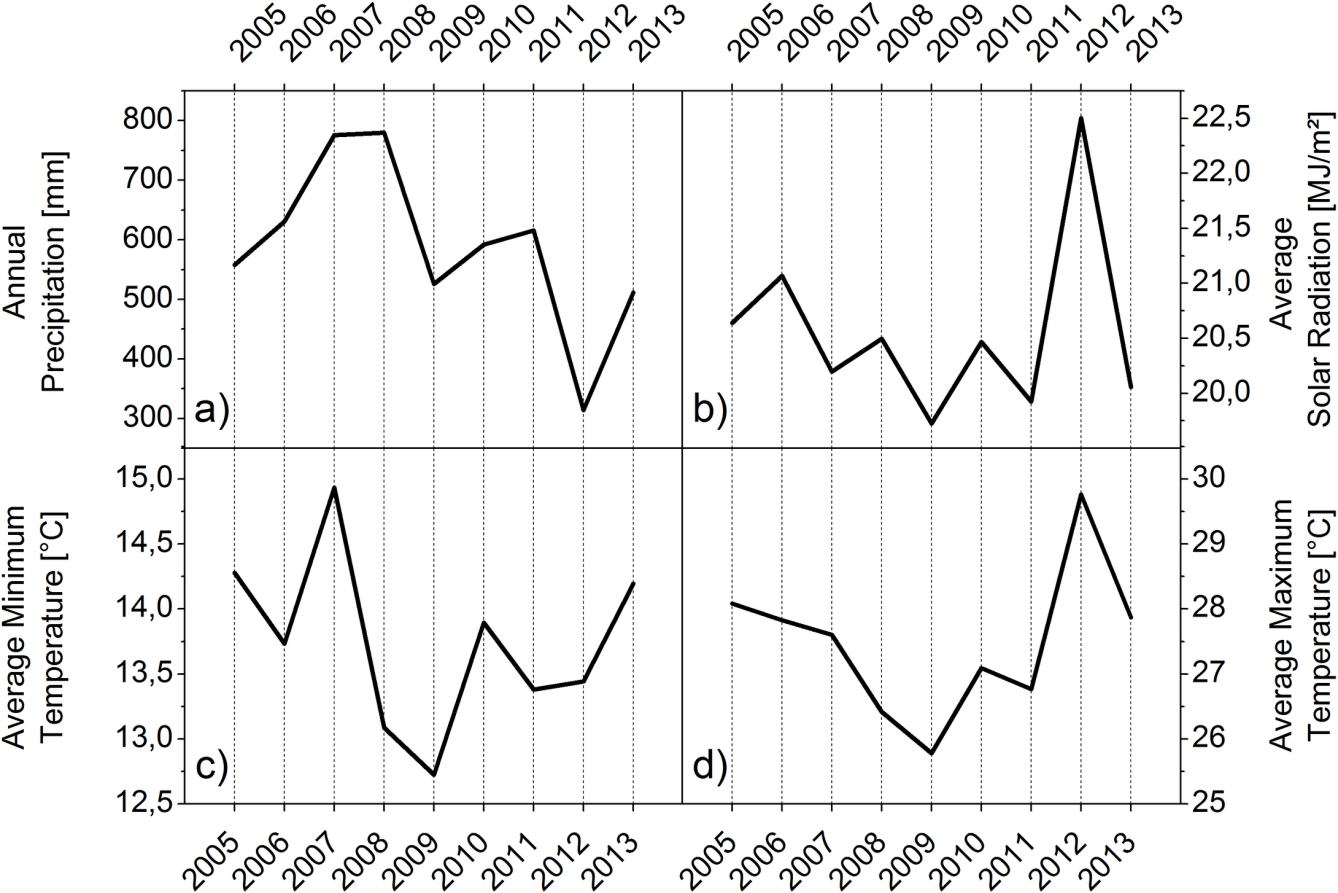
Annual weather data recorded in the entire study area between 2005 and 2013. The figures illustrate the annual sum of precipitation (a) as well as seasonal average solar radiation (b), minimum temperature (c) and maximum temperature (d). The seasonal average includes recorded data from May 1^st^ to September 30^th^ of the respective year for all weather stations in Fig 1.

With the help of weather variables between 2005 and 2013 presented in Fig 4 large parts of the variations of the total water supply, yields and the dynamics of WP can be explained. In 2012 total water supply amounts were average. However, the large irrigation amounts in 2012 stand out from all other years. Also the yield in 2012 were above average in both NRDs. This is particularly interesting since in 2012, Nebraska was experiencing a strong drought. The weather variables of the study area shown in Fig 4 illustrate the unfavorable weather condition for crop growth in this year. Total precipitation amounts in 2012 dropped to only 314 mm and solar radiation and maximum temperature measurements in the growing season were by far the highest of all the years studied. However, farmers in the study area had enough irrigation water available to compensate for the lack of precipitation. In addition, the highly developed irrigation technology commonly used in the study area can provide better control over the amounts of water applied to the crops and therefore farmers can adjust water amounts more flexibly in order to generate better growing conditions. Furthermore, irrigation has an evaporative cooling effect, which further compensates for the drought impacts by decreasing temperatures and thus extending the duration of the grain filling, which gives the plant more time to accumulate biomass [29]. The relationship between temperature and crop development is described in the concept of thermal time, which is explained in more detail below. These conditions resulted not only in high yields in the study area but also a very high WP value in the Tri Basin. In the Central Platte the very high irrigation amounts in 2012 resulted in a WP value that was slightly below the WP value compared to the previous and following year but still above average.

The highest actual WP value in the Tri Basin was recorded in 2009. In this year yields were also the highest in the Tri Basin, while in the Central Platte they were above average. Total water supply amounts as well as irrigation amounts in 2009 were on average in both NRDs. In the temperature charts presented in Fig 4, it is noticeable that both seasonal maximum and minimum temperatures and also solar radiation values in 2009 were the lowest of all examined years. With the help of the Hybrid-Maize results, which simulated highest WP values and yields in 2009, it was possible to identify factors favoring corn production in this year. The length of the simulated growing season at each location was by far the longest in 2009. This was possibly due to the cooler temperatures, which resulted in more days until the required accumulated heat units for crop maturity were reached. Considering the concept of thermal time crops reach maturity after a specific number of ‘growing degree days’ expressed by the sum of daily temperature values [30]. The definition includes a base temperature below which crop development does not progress [31]. For maize it is common to use a base temperature of 10°C [32]. When daily temperatures are relatively low but still above the base temperature, the plants have more days to intercept light for biomass accumulation and thus develop higher yields [29].

In the Central Platte, WP is the lowest in 2008. This is mainly the result of above average total water supply amounts and low yields. The precipitation amounts, which are the highest of all analyzed years reveal the reason of high total water supply amounts in that year, which negatively influences WP. The high precipitation amounts could also be the reason for a slight decline in yields. According to several authors it might be possible that nutrients leached from the root zone through percolation and runoff due to strong rainfall [5, 33]. This assumption is supported by the HYDRUS-1D simulation results, which demonstrate the deeper movement of nitrate in the root zone at several locations with different soil conditions in 2008 in contrast to the dry year 2012 (S2 Fig).

The examination of the relationship of yields, amounts of water supply and irrigation with weather variables show that weather conditions have a big impact on the variations of WP. Furthermore, the results demonstrate how farmers’ water management decisions can alter WP. Differences in actual annual WP values can be related to prevailing weather conditions. If water amounts are too high due to extreme precipitation events it is hard for farmers to compensate for this condition, and the yield might suffer. A lack of water however, would naturally result in unfavorable conditions for plant growth but this can be compensated by farmers through increasing irrigation, thus securing higher yields, especially where sufficient water is available. Nevertheless, large amounts of irrigation can also decrease WP despite high yields. In the Tri Basin WP values were among the highest in the drought year of 2012. However, in the Central Platte very high irrigation amounts–average of 623 mm in 2012 did not lead to the highest WP value of the observation period although yields were very high.

### Impact of weather conditions on crop water requirement

**Fig 5:**
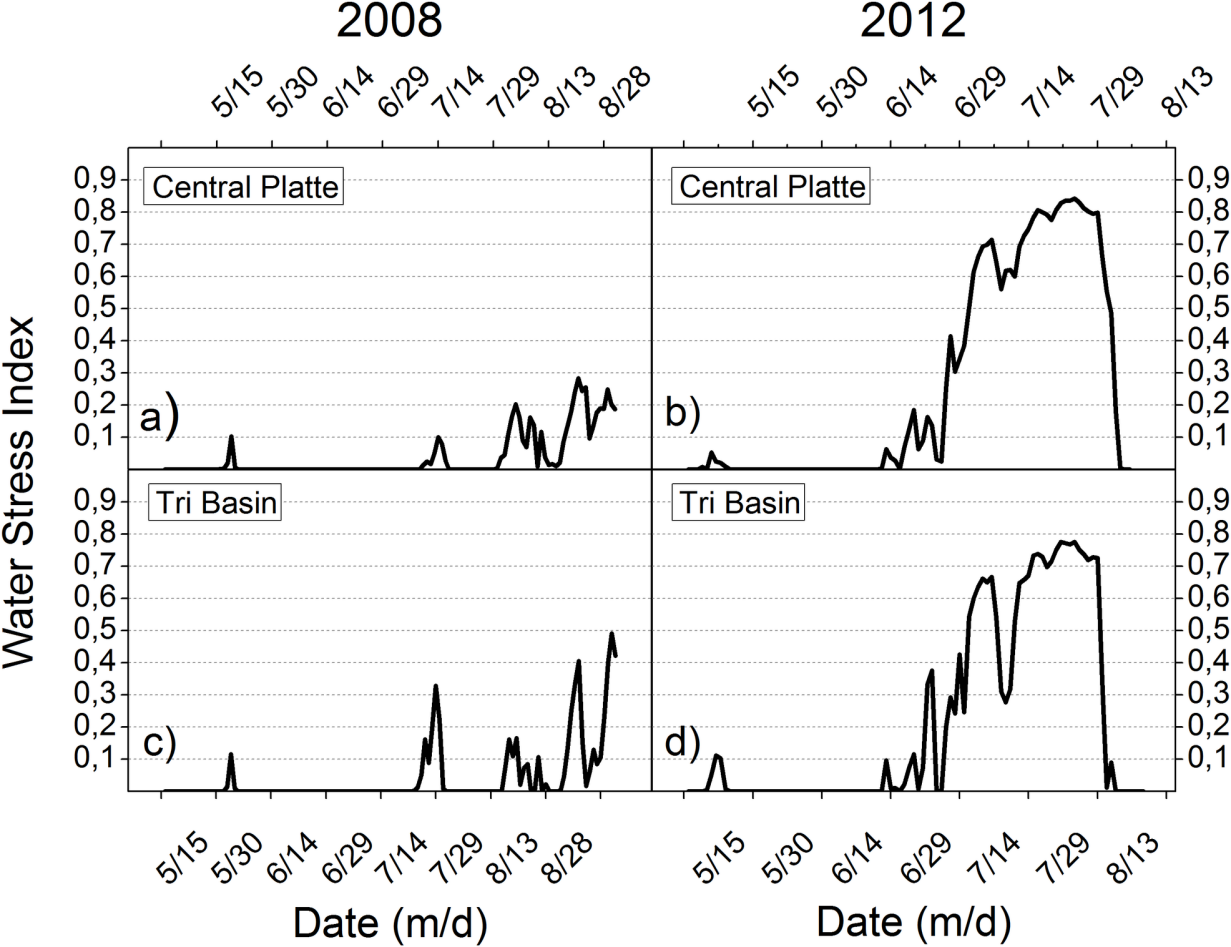
Water Stress Index derived from the daily simulated gap between crop water demand and actual crop water consumption. The simulation results were generated with Hybrid-Maize assuming non-irrigated conditions. Water Stress of the Central Platte (a, b) and the Tri Basin (c, d) is compared between the wet year 2008 and the dry year 2012.

The daily simulation results of Hybrid-Maize can be used to analyze the different seasonal courses of water stress, as illustrated in Fig 5, resulting from changing crop water needs related to biomass growth and the present soil water balance. The presented daily simulation results in Fig 5 assume no irrigation to compensate for crop water stress. Comparison of the driest (2012) and the wettest (2008) years between 2005 and 2013 demonstrate the necessity of adapting water management to both the seasonal changing crop water needs and annual changing environmental conditions. In 2008 the frequent rainfall nearly met crop water needs until the end of the season, illustrated by a very low crop water stress index. However, in 2012 large irrigation amounts were necessary to prevent crop water stress, especially in the second half of the growing season. These results show how irrigation needs to be temporally adjusted in order to meet crop water needs to gain high yields. Additionally, they demonstrate how to save irrigation water if properly adapting to seasonal and annual changing crop water needs and weather conditions. Considering this can lower irrigation water usage and thus increase WP.

### Impact of fertilizer supply on water productivity

The decreased WP and the simulated lower amounts of nutrients in the root zone due to strong rainfall in 2008 indicates the influence of nutrient availability on WP. Similar to crop water needs, crop nutrient needs vary during the course of the season. In addition, the varying availability of nutrients influenced through leaching processes caused by high rainfall or excessive irrigation needs to be considered. In this respect, water management should be coordinated with nutrient management, since the application of nutrients prior to irrigating the field can have the same effect on vertical nutrient transport in the soil as strong rainfall. Furthermore, coordinating the application of fertilizer with rainfall and irrigation in order to prevent leaching of nutrients beyond the root zone can lead to a reduced need for fertilizers. This could help lower nitrate contamination of ground water, which is an issue at the studied locations. In large parts of the study area nitrate concentrations in the groundwater are higher than the safe drinking water standard of 10 mg/L determined by the United States Environmental Protection Agency [34].

A comprehensive analysis of WP in order to improve the sustainability of agricultural water management should take into account the consequences, which can result from increasing yields per unit of increased water supply. Increasing the availability of fertilizer might improve WP but could come at the expense of the environment. Considering this, WP was analyzed in this study while also evaluating N-fertilizer application. In this respect, the analysis with Maize-N revealed that at 82% of the analyzed locations more N was being added between the years 2005 and 2013 than necessary for the observed yields (S3 Fig). Optimal application rates of N, which still provide high yields and thus do not decrease WP while not causing environmental damage should be the goal. In this respect, seasonal N absorption by plants and leaching processes through percolating water should be studied further. Particularly, applying mid to late season nitrogen via sprinkler irrigation system will be most beneficial. Thus, strategies for coordinating water and nutrient management can be developed in order to grow more crops per drops without impairing groundwater quality.

### Impact of groundwater levels on Water Productivity

So far WP was defined by the amount of grain yield per unit of total water supply. The concept of WP would also allow defining WP by the net benefit of corn yields per unit of total water supply. Thereby, economic parameters representing the variations of environmental conditions can be added. Thus, groundwater level fluctuations, which have an impact on energy costs for pumping irrigation water can be included in an economic WP coefficient. A decline in groundwater levels would result in more irrigation costs due to growing energy costs to pump irrigation water from increasing depths. This would reduce the net benefit of corn yields per amount of irrigation water used and thus result in a lower economic WP. Examples in Nebraska with large groundwater decline demonstrate this relationship between economic WP and groundwater levels (S4 Fig). The economic WP values in these examples were calculated with the average corn yield production and water use amounts as well as corn and energy prices recorded in the observed areas between 2005 and 2013. The only changing variable is the groundwater level recorded in July between 1970 and 2015 at both locations.

These additional examples and the demonstrated impact of weather variables show the variety of factors influencing WP. The many parameters influencing yields and water use result in a temporal dynamic WP, which needs to be considered when the productivity of water use is evaluated. In addition, the definition of WP needs to be clear, based on the purpose of the WP analysis. Including economic factors like pumping costs can help to form incentives to improve WP. Additional parameters like this can further increase the scope of a WP analysis and help to generate suggestions for improvements.

## Conclusion

Based on the simulation results with Hybrid-Maize, improving WP is possible in large parts of the study area. Yields in the analyzed fields are already at a high level and thus no crucial gaps between simulated optimal and actual yields could be observed. However, irrigation amounts used on the majority of the analyzed fields exceeded the necessary amounts to achieve optimal yield rates. Due to excessive irrigation, most WP values do not achieve ideal levels.

With the available data it is not possible to draw conclusions why farmers decide to use too much water for irrigation. Farmers might know about the lower irrigation requirements for optimal yields but because of abundant water supply in the study area they do not have an incentive to limit irrigation water. Furthermore, they might avoid the risk of yield reduction caused by unfavorable weather conditions. The potential impact of weather variables on yields and WP are hard to predict in advance and therefore farmers might apply more water to avoid possible deficit in soil water balance, which would decrease yields. For example, the high yields and the above average WP values of the drought-year 2012 demonstrates that water management supported by advanced irrigation techniques and abundant water resources can compensate for unfavorable weather conditions. In contrast to that, strong rainfall in 2008, which could not be compensated for through agricultural management, illustrate the dependency of WP on weather conditions. This dependency is partially responsible for annual variations of WP following year-to-year dynamics of weather parameters.

The application of fertilizers additionally influences differences in corn yields and WP while also affecting environmental quality. Therefore, the analysis of WP should consider the side effects of improving yields per unit of water. The simulation results of this study revealed that the application of N-fertilizers could be reduced in most studied locations and years without decreasing yield and WP. Due to groundwater contamination with nitrate this should be especially considered in the study area. Furthermore, the simulated leaching of nutrients illustrates the need for adjusting timing of fertilizer application to varying weather conditions as well as to water application. Rainfall and irrigation water can transport nutrients beyond the root zone and thus reduce the availability to crops. This can lead to reduced nutrient availability and a decreased WP, as well as to the deterioration of groundwater quality.

In summary, the results of this study illustrate the year-to-year variations of WP based on a range of factors influencing crop growth and water use. In particular, the annual variations of weather variables influence growing conditions and water needs of crops and thus WP. Although conditions to optimize WP can be altered by irrigation and fertilizer management, the year-to-year variations of WP still show a large dependency on annual weather conditions. Therefore, the evaluation of just a single year is not suitable to capture the whole scope of WP at a location. In order to draw a conclusion about WP, the variations of yields per unit of water need to be considered in the evaluation. In this study nine consecutive years were analyzed, from which the five years between 2008 and 2012 show most characteristics to describe the factors influencing the variations of WP. Furthermore, the results of a WP analysis should be considered in the context of an agricultural system with many interdependencies. In this respect, an additional analysis of the relation between environmental quality and increasing crop yields, or on economic incentives for farmers to decrease water use can support the search for methods to change WP in an area.

As with any studies that are based on model simulations, potential errors and uncertainties in the simulation results need to be considered and better quantified in the future, although both the Hybrid-Maize and Maize-N model were developed in this area and have been tested positively. HYDRUS-1D simulations were based on limited amount of information and no geochemical reactions were considered. For better accuracy, nitrogen fate and other geochemical reactions need to be considered.

## Supporting Information

S1 Dataset Overview of processed data. (XLSX)

S1 Fig Outliers outside the range of the triple standard deviation of the observed data. (TIF)

S2 Fig Simulated vertical distribution of solutes in soil profiles at different locations in the study area. The simulations were conducted at selected locations with different soil types including Clay (a, b); Clay Loam (c, d); Silt Loam (e, f) and Sandy Loam (g, h). The results illustrate the differences in solute transport between the wet year 2008 and the dry year 2012 at each location.
(TIF)

S3 Fig Simulated optimal nitrogen supply and actual nitrogen supply. Optimal nitrogen supply was simulated for the observed yield amounts in the study area (n = 206) and compared with the actual nitrogen supply per observed yield amounts (n = 206).
(TIF)

S4 Fig The impact decreasing groundwater on economic WP measured in Corn Yield [$] per amount of water [m3]. Energy costs for pumping irrigation water using diesel, electricity or natural gas are subtracted from corn yield values. Groundwater levels were measured in July between 1970 and 2015 at two wells in Buffalo County (a, b, c) and in Dundy County (d, e, f).
(TIF)
