# Supplementary figures and images for: Temporal Variations of Water Productivity in Irrigated Corn: An Analysis of Factors Influencing Yield and Water Use across Central Nebraska
Source: PLoS One. 2016 Aug 30;11(8):e0161944. doi: 10.1371/journal.pone.0161944 (PMC5004851; doi:10.1371/journal.pone.0161944)

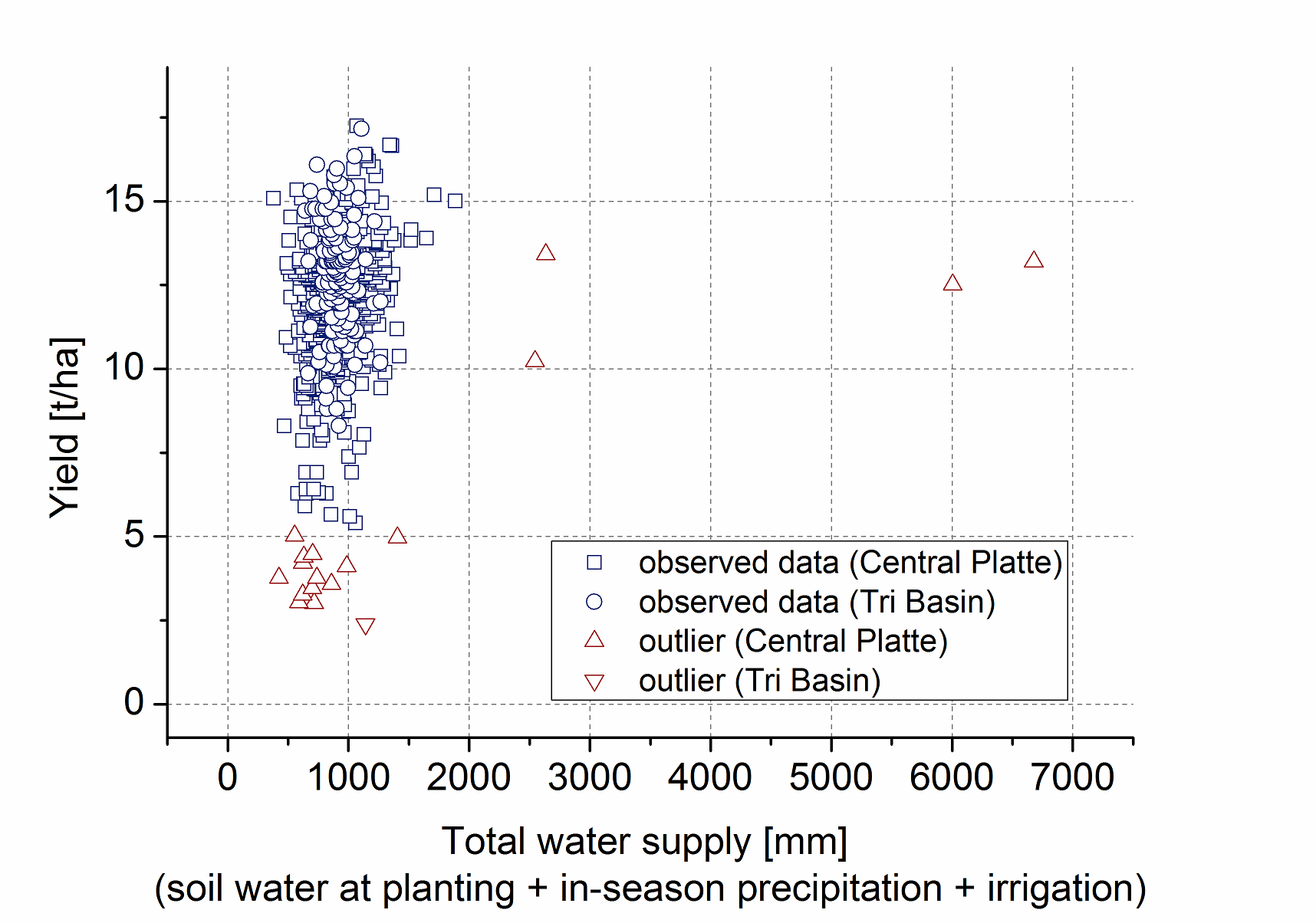

Supplement: S1 Fig — (TIF) [file pone.0161944.s002.tif]

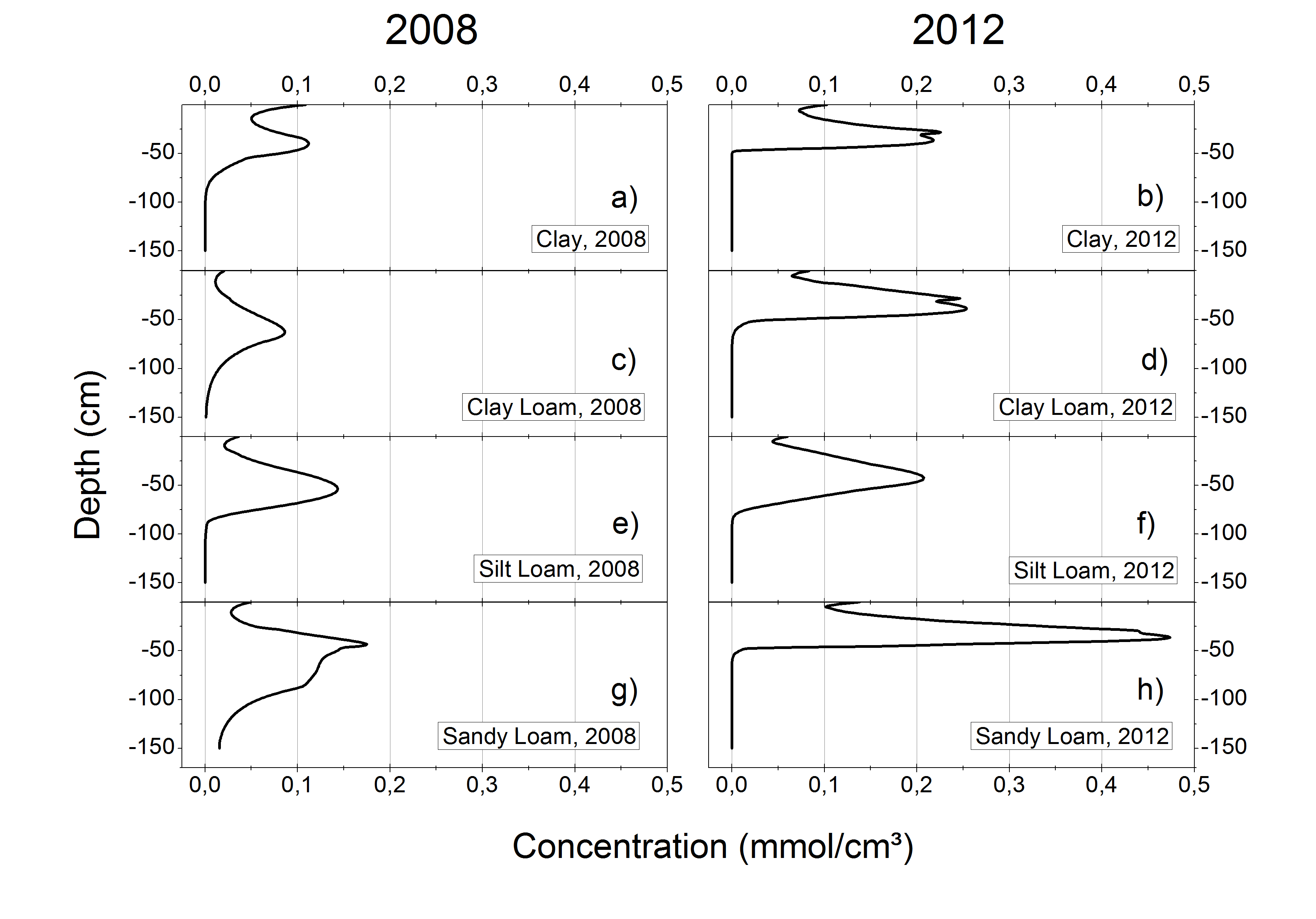

Supplement: S2 Fig — The simulations were conducted at selected locations with different soil types including Clay (a, b); Clay Loam (c, d); Silt Loam (e, f) and Sandy Loam (g, h). The results illustrate the differences in solute transport between the wet year 2008 and the dry year 2012 at each location. (TIF) [file pone.0161944.s003.tif]

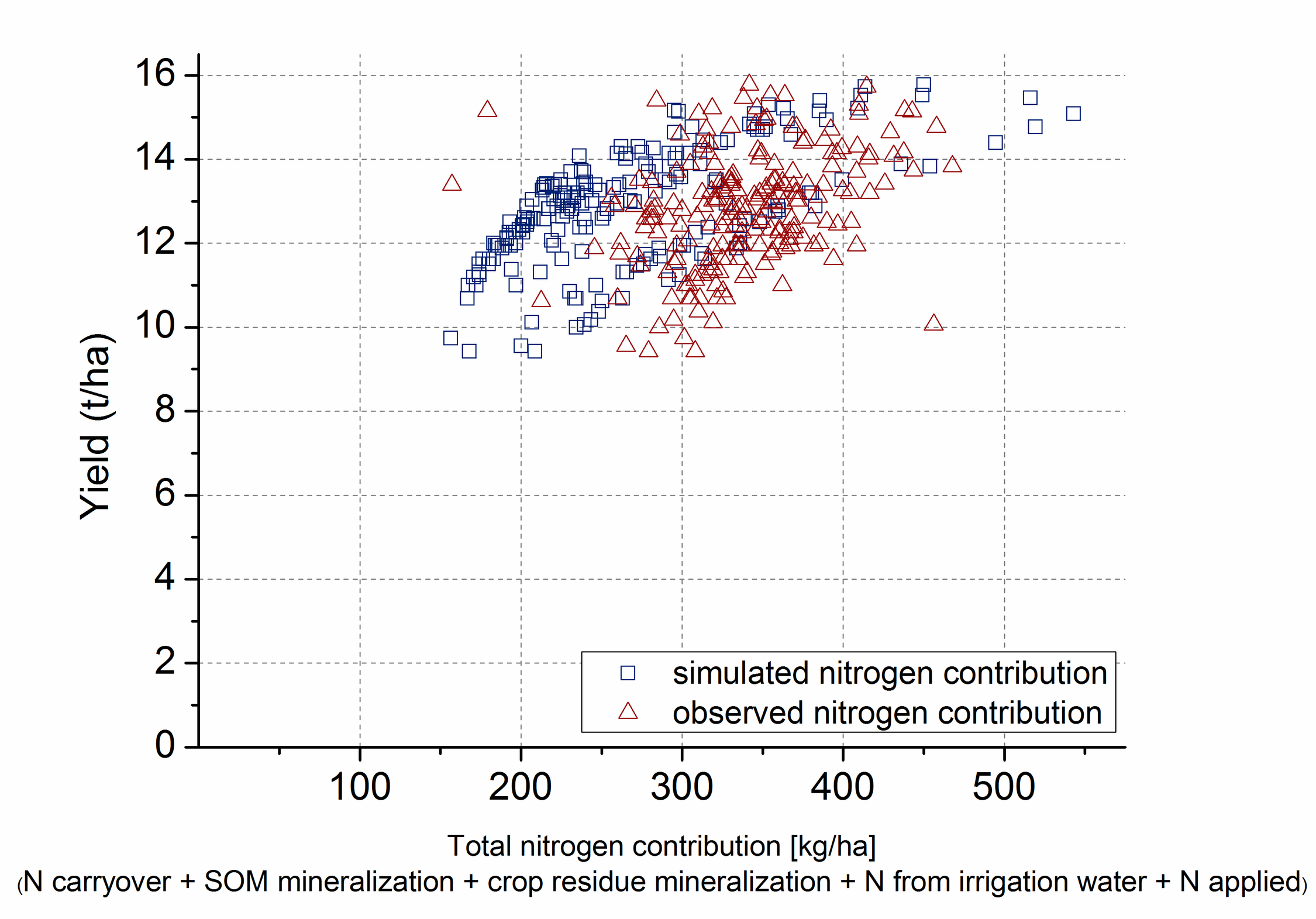

Supplement: S3 Fig — Optimal nitrogen supply was simulated for the observed yield amounts in the study area (n = 206) and compared with the actual nitrogen supply per observed yield amounts (n = 206). (TIF) [file pone.0161944.s004.tif]

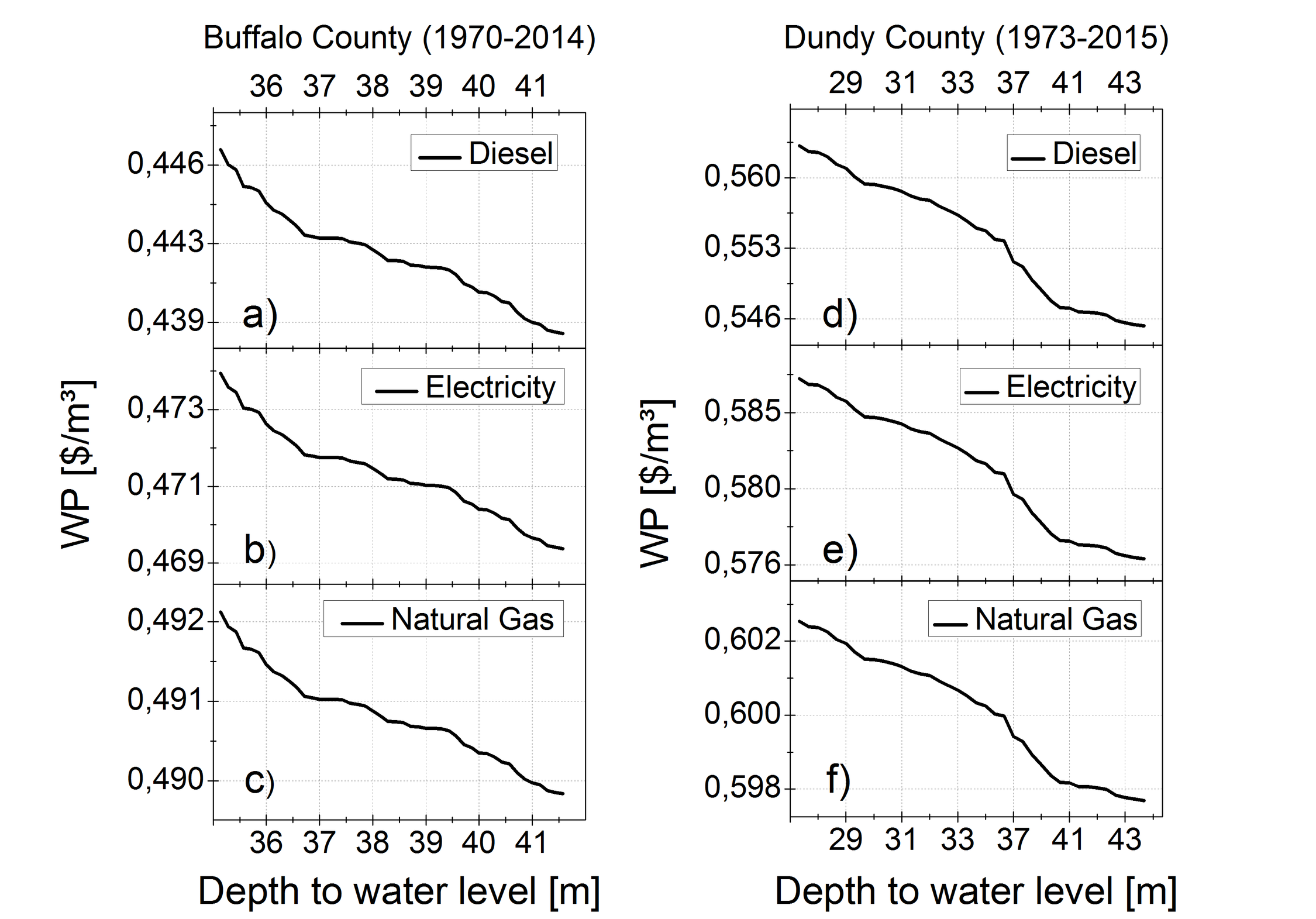

Supplement: S4 Fig — Energy costs for pumping irrigation water using diesel, electricity or natural gas are subtracted from corn yield values. Groundwater levels were measured in July between 1970 and 2015 at two wells in Buffalo County (a, b, c) and in Dundy County (d, e, f). (TIF) [file pone.0161944.s005.tif]
